# PTEN Increases Autophagy and Inhibits the Ubiquitin-Proteasome Pathway in Glioma Cells Independently of its Lipid Phosphatase Activity

**DOI:** 10.1371/journal.pone.0083318

**Published:** 2013-12-13

**Authors:** Rajaa Errafiy, Carmen Aguado, Ghita Ghislat, Juan M. Esteve, Anabel Gil, Mohammed Loutfi, Erwin Knecht

**Affiliations:** 1 Laboratorio de Biología Celular, Centro de Investigación Príncipe Felipe, Valencia, Spain; 2 Centro de Investigación Biomédica en Red de Enfermedades, Raras Valencia, Spain; 3 Laboratoire de Biochimie et Biologie Moléculaire, Faculté des Sciences, Ain chock, B.P 5366, Casablanca, Morocco; NIH/NCI, United States of America

## Abstract

Two major mechanisms of intracellular protein degradation, autophagy and the ubiquitin-proteasome pathway, operate in mammalian cells. *PTEN*, which is frequently mutated in glioblastomas, is a tumor suppressor gene that encodes a dual specificity phosphatase that antagonizes the phosphatidylinositol 3-kinase class I/AKT/mTOR pathway, which is a key regulator of autophagy. Here, we investigated in U87MG human glioma cells the role of PTEN in the regulation of autophagy and the ubiquitin-proteasome pathway, because both are functionally linked and are relevant in cancer progression. Since U87MG glioma cells lack a functional PTEN, we used stable clones that express, under the control of a tetracycline-inducible system (Tet-on), wild-type PTEN and two of its mutants, G129E-PTEN and C124S-PTEN, which, respectively, lack the lipid phosphatase activity only and both the lipid and the protein phosphatase activities of this protein. Expression of PTEN in U87MG glioma cells decreased proteasome activity and also reduced protein ubiquitination. On the contrary, expression of PTEN increased the autophagic flux and the lysosomal mass. Interestingly, and although PTEN negatively regulates the phosphatidylinositol 3-kinase class I/AKT/mTOR signaling pathway by its lipid phosphatase activity, both effects in U87MG cells were independent of this activity. These results suggest a new mTOR-independent signaling pathway by which PTEN can regulate in opposite directions the main mechanisms of intracellular protein degradation.

## Introduction

Macroautophagy, hereafter called autophagy, and the ubiquitin-proteasome system (UPS) are the main pathways of intracellular protein degradation in mammalian cells [1]. Autophagy is essentially responsible for the degradation under starvation conditions of long-lived proteins, cytoplasmic organelles and other cell components. The initial step is the formation of a double membrane structure, called the phagophore, which surrounds cytoplasmic material and closes to form a double membrane autophagosome. Then, the autophagosome fuses with endosomes and lysosomes to form the single membrane autolysosome with hydrolytic enzymes that degrade its content [2,3]. In contrast to autophagy, which is less selective and more adapted to cell survival under starvation, the UPS is highly selective and mainly involved in the prompt degradation of abnormal, improperly assembled and other short-lived proteins, thus controlling both protein quantity and quality and playing a crucial role in the survival of eukaryotic cells under basal conditions [4]. The UPS works in two main steps. First, various ubiquitin molecules are attached to a protein substrate through specific ligases (E3) in concert with other enzymes (E1 and E2) and, next, the polyubiquitinated protein is deubiquitinated to release reusable ubiquitin molecules and the remaining protein is degraded by the 26S/30S proteasome [5,6].

Both autophagy and the UPS play essential roles in cellular protein homeostasis and control many cell functions, including DNA repair and transcription, mRNA translation, cell growth and proliferation, apoptosis and the immune response [1]. Therefore, defects in their functions have been implicated in several human pathologies, including cancer [7,8]. For example, the regulation of autophagy frequently overlaps with signaling pathways involved in tumorigenesis [9,10], especially with the phosphatidylinositol 3-kinase (PI3K) class I/AKT/mTOR pathway. 

An upstream regulator of mTOR is the tumor suppressor gene *PTEN* (phosphatase and tensin homologue deleted on chromosome 10), which is mutated or deleted in a great number of human tumors, including gliomas, the most aggressive type of primary brain tumors [11,12]. This gene codifies a dual specificity phosphatase [13,14] and its best known biological role resides in the lipid phosphatase activity that antagonizes the activity of the PI3K class I oncoprotein, which is required for the phosphorylation and activation of the proto-oncogene protein kinase B/AKT [15,16]. This results in several effects, including promotion of apoptosis and inhibition of cell cycle progression [17]. However, there are also PTEN functions beyond this specific lipid phosphatase role [18]. 

Although an activation of autophagy by PTEN through its classical dephosphorylation activity of phosphatydilinositol (3,4,5)-trisphosphate has been described in human colon cancer HT29 cells [19], a role of PTEN in the regulation of the UPS is less established. This is an important issue, because the UPS and autophagy are thought to cooperate, each regulating the other [20-22]. Also, it is unknown if PTEN has some effect on the main mechanisms of intracellular protein degradation independently of its lipid phosphatase activity. Therefore, we have examined here the possible regulation of autophagy and the UPS by the two purported (lipid and protein) phosphatase activities of PTEN under high and low proteolysis conditions in U87MG human glioma cells that lack a functional PTEN. We report that both intracellular protein degradation systems become affected by the conditional expression of PTEN in opposite directions and that, surprisingly, in U87MG human glioma cells these effects, including the activation of autophagy, are mainly independent of the lipid phosphatase activity of PTEN. 

## Results

**Figure 1:**
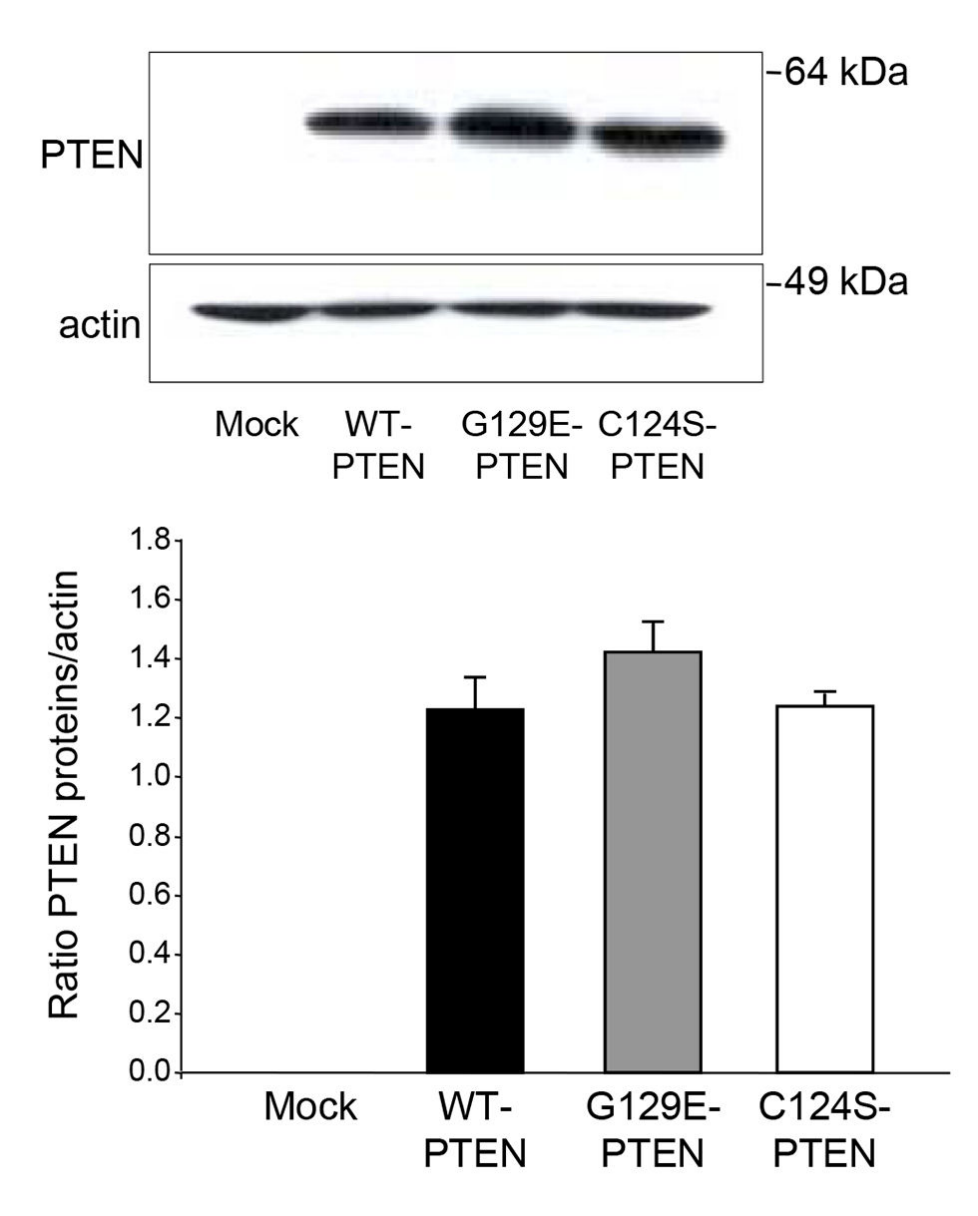
Expression levels of PTEN in various clones of U87MG cells. U87MG human glioma cells, mock-treated or expressing WT-PTEN or two of its mutants (G129E-PTEN and C124S-PTEN) under the control of a tetracycline-inducible system, were incubated in full DMEM and treated with doxycycline as described in Materials and Methods. After 18 h, expression of PTEN was analyzed by Western blot in whole lysates using anti-PTEN and, as a loading control, anti-actin antibodies. Molecular weight markers are indicated on the right. PTEN and actin bands were quantified by densitometry and their ratios are shown in the histogram below. Results are means ± SD of three independent experiments. No significant differences were found among the cells that express WT-PTEN or its two mutants.

PTEN is not expressed in U87MG glioma cells and ectopic expression of WT-PTEN in these cells results in cell death, as it has been also described in other cancer cell lines [23]. Therefore, we used stable clones that express WT-PTEN or its two mutants, G129E-PTEN (lipid phosphatase inactive) and C124S-PTEN (protein and lipid phosphatase inactive), all under the control of a tetracycline-inducible (Tet-on) system (see Materials and Methods). After induction of their expression, the levels of the various PTEN proteins in the clones that were used in the following experiments were found to be comparable (Figure 1).

### PTEN expression in U87MG glioma cells inhibits the ubiquitin-proteasome system

**Figure 2:**
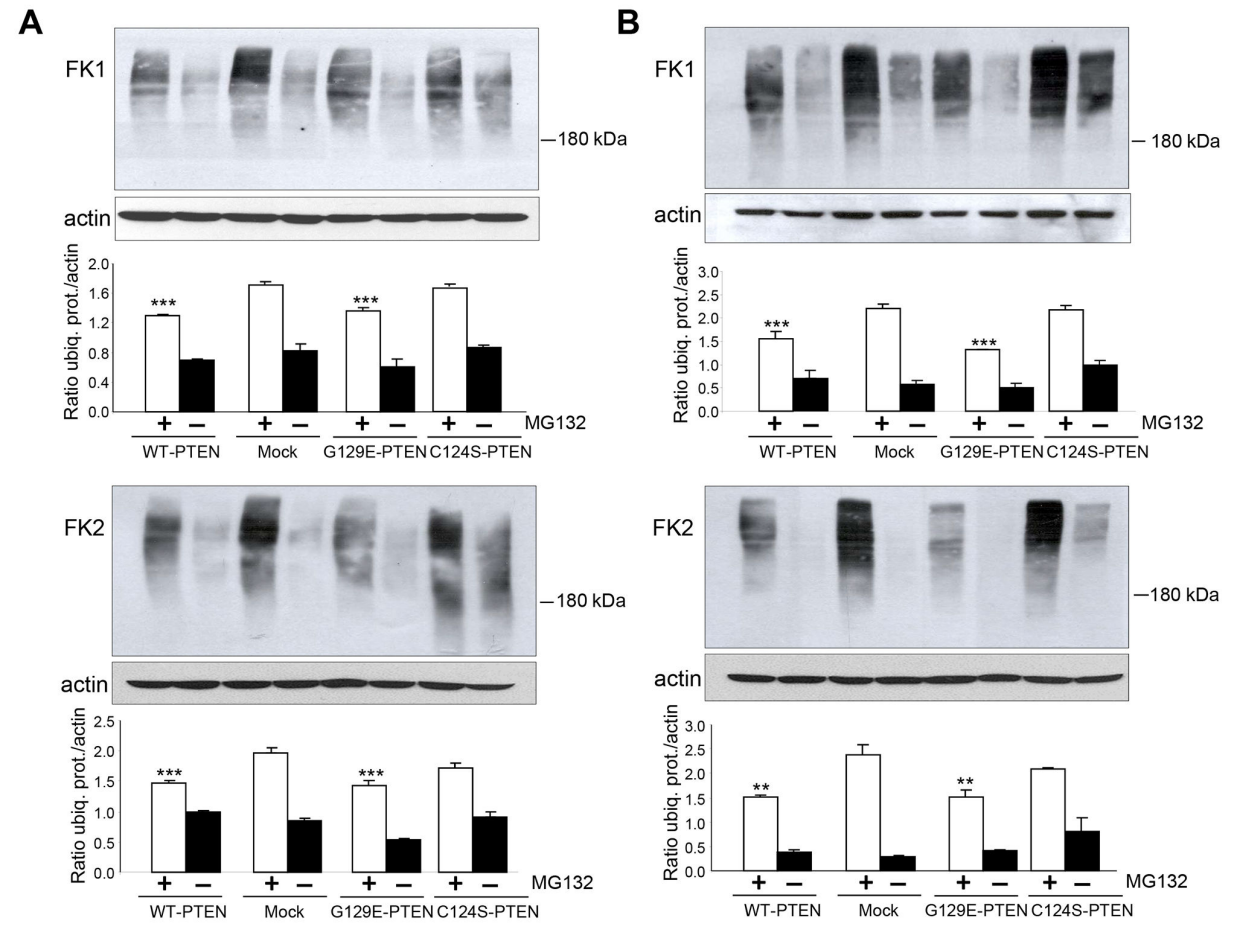
Decreased levels of ubiquitinated proteins in U87MG cells that express PTEN. The same U87MG cells from Figure 1 were incubated for 3 h in KH without (**A**) or with (**B**) amino acids and insulin. Where indicated, the cells were treated with the proteasome inhibitor MG132 (50 µM). Extracts (75 µg protein) of the cells were subjected to SDS-PAGE and analyzed by Western blot using two different antibodies that recognize ubiquitinated proteins (FK1 and FK2) and, as a loading control, an antibody that recognizes actin. The amount of ubiquitinated proteins (ubiq. prot.) was quantified by densitometry and normalized to the levels of actin. Results, which are means ± SD of three independent experiments, are shown below the representative Western blots. Differences from the corresponding values (with or without MG132) in mock-treated cells were found to be significant at **p<0.01 and ***p<0.005, respectively.

UPS activity can be determined both at the polyubiquitination step and by measuring the chymotrypsin-like activity of proteasomes, which is the most important in intracellular protein degradation [24]. Polyubiquitination is the first step in the degradation of proteins by proteasomes and, therefore, we started investigating the effect of PTEN expression in U87MG cells on the levels of polyubiquitinated proteins. Once polyubiquitinated, the proteins are almost immediately degraded by proteasomes. Therefore, we used the proteasome inhibitor carbobenzoxyl-leucinyl-leucinyl-leucinal (MG132) to specifically investigate the ubiquitination step. Two different antibodies (FK1 and FK2, see Materials and Methods) were used to detect ubiquitinated proteins under both high (Figure 2A, incubation in KH alone, see Materials and Methods) and low (Figure 2B, incubation in KH plus insulin and amino acids) proteolysis conditions. As expected, in both conditions (Figure 2A and B, odd lanes) more ubiquitinated proteins accumulated in the presence than in the absence of MG132. In addition, the levels of ubiquitinated proteins were slightly lower in the cells expressing WT-PTEN than in the mock-treated U87MG cells. Interestingly, in the cells that express the G129E- or C124S-PTEN mutants, the levels of polyubiquitinated proteins resembled, respectively, those in the cells that express WT-PTEN or in the mock-treated cells. 

**Figure 3:**
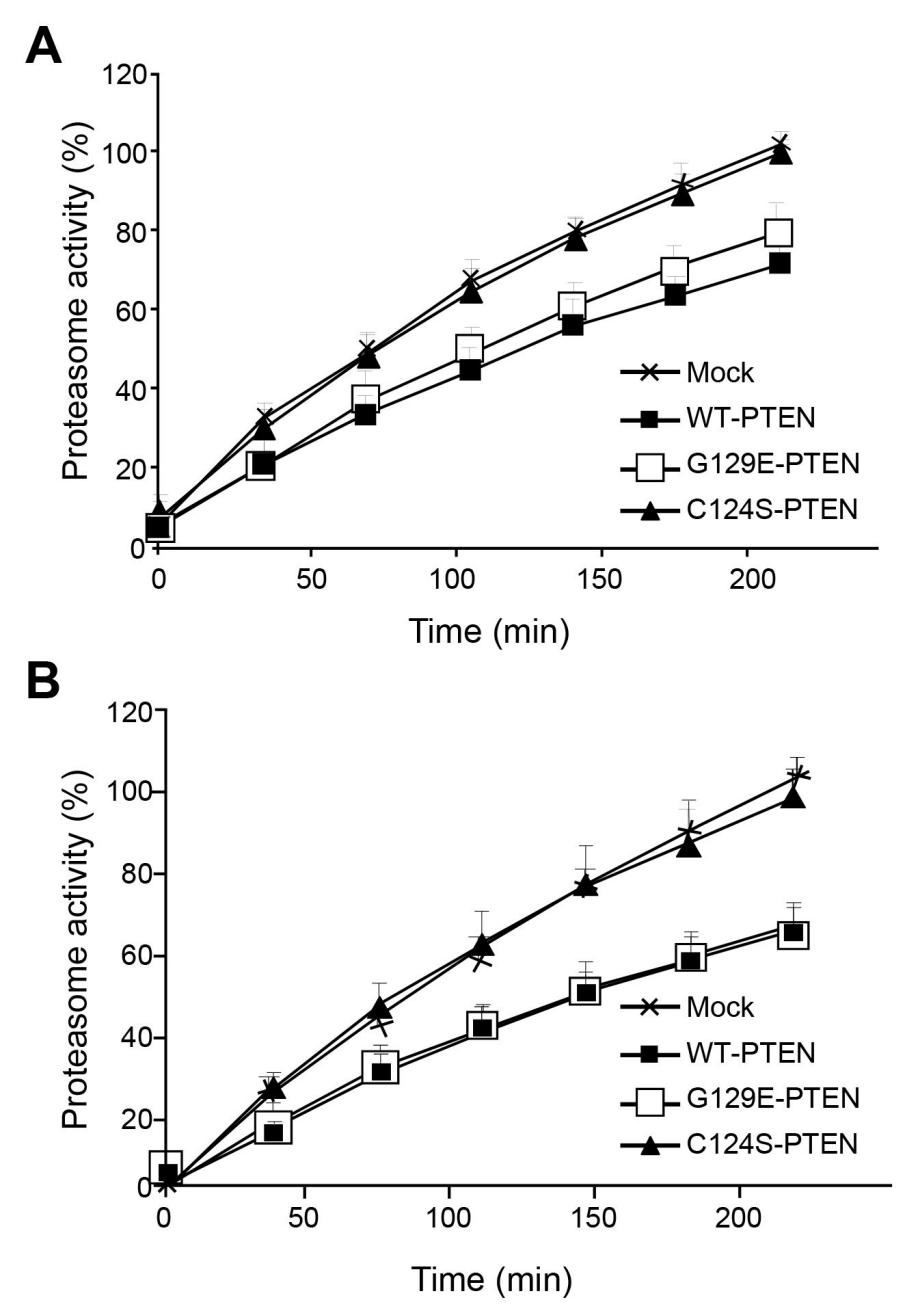
Decreased chymotrypsin-like activity of proteasomes in U87MG cells that express PTEN. The same cells from Figure 2 were cultured in KH without (**A**) or with (**B**) amino acids and insulin. The chymotrypsin-like peptidase activity of proteasomes was determined using the fluorogenic substrate N-Suc-LLVY-AMC as described in Materials and Methods. As a control, the specific proteasome inhibitor lactacystin (40 µM) was added and the activity of proteasomes was determined as the difference obtained in the absence and in the presence of lactacystin, which was about 12-15% of the activity measured without the inhibitor. Results are expressed in percentage relative to the fluorescence values of the mock-treated cells at 3.5 h and are means ± SD of five independent experiments, each of them run in duplicate.

Next and to evaluate if PTEN expression also affects the other step of the UPS, we measured in the same cells under high and low proteolysis conditions the chymotrypsin-like peptidase activity of proteasomes with the fluorogenic substrate N-Suc-LLVY-AMC. As shown in Figure 3A, expression of WT-PTEN under high proteolysis conditions decreased the chymotrypsin-like activity of proteasomes and, again, this decrease was also observed in the cells that express the G129E-PTEN mutant but not in those that express the C124S-PTEN mutant. Similar results were obtained under low proteolysis conditions (Figure 3B).

Thus, it appears that PTEN inhibits somehow the UPS at both the ubiquitination and the proteolytic steps. Moreover, since C124S-PTEN lacks the lipid and protein phosphatase activities, while G129E-PTEN only lacks the lipid phosphatase activity of PTEN, these effects appear to be independent of the lipid phosphatase activity of PTEN. 

### PTEN expression in U87MG glioma cells increases autophagy

**Figure 4:**
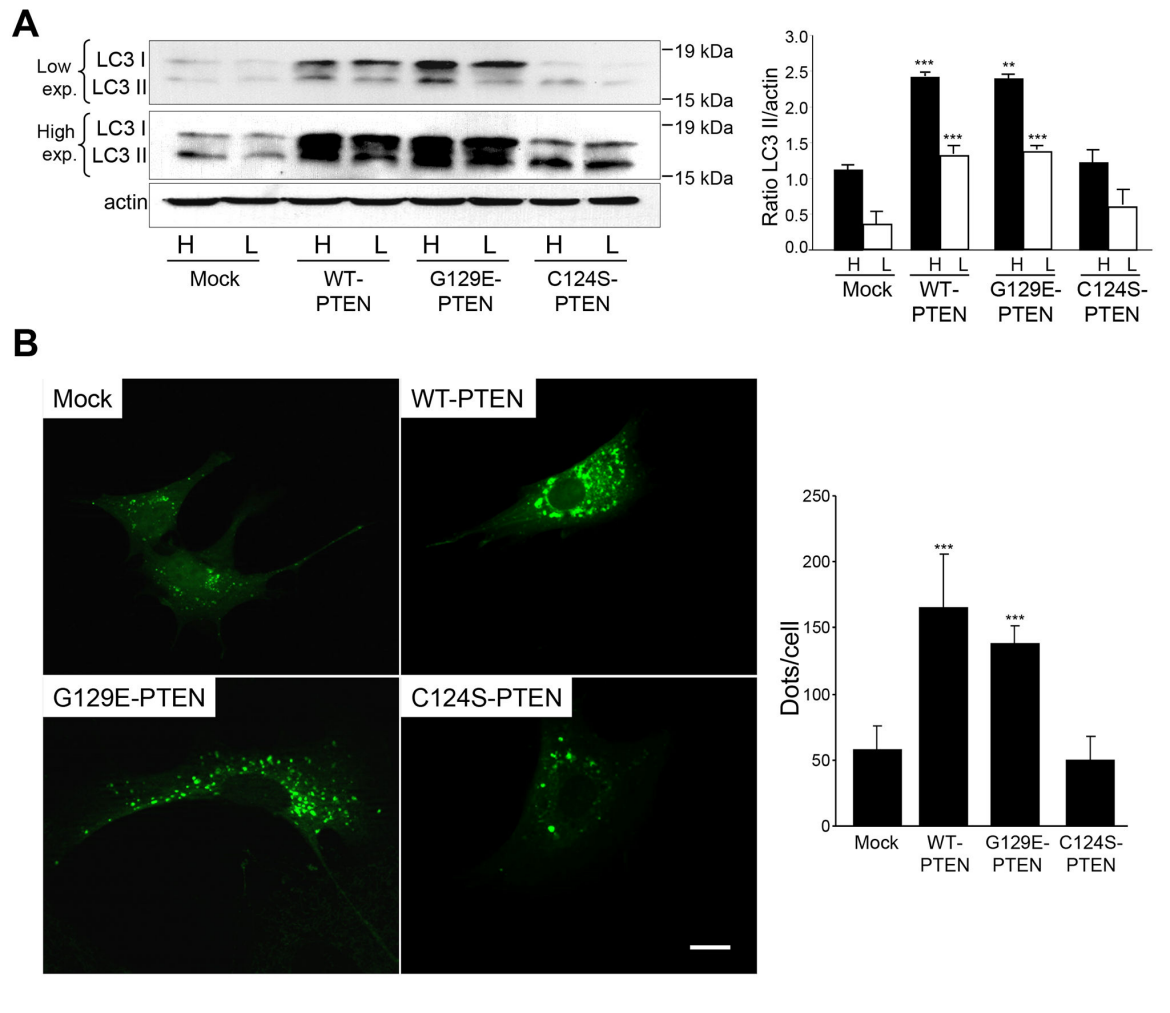
PTEN expression in U87MG cells increases the formation of autophagosomes. **A**) U87MG cells expressing WT-PTEN, C124S-PTEN and G129E-PTEN or mock-treated were incubated in high (H) or low (L) proteolysis media (see Materials and Methods) for 2 h in the presence of lysosomal inhibitors (100 µM leupeptin and 20 mM NH_4_Cl). Extracts (75 µg protein) were analyzed by SDS-PAGE and immunoblot, with low and high exposure (exp.), using an antibody against LC3 and, as a loading control, an antibody that recognizes actin. A representative experiment is shown. The position of LC3-I and LC3-II bands are indicated on the left and molecular weight markers are indicated on the right. The histogram on the right shows the means ± SD of the densitometric analysis of the LC3-II/actin ratios from five different experiments. Stars indicate statistically significant differences from the corresponding (high or low proteolysis conditions) values in mock-treated cells at **p<0.01 and ***p<0.005. **B**) Representative fluorescent images of the indicated cell lines transitorily expressing EGFP-LC3 (at 48 h post-transfection) and incubated under high proteolysis conditions for 2 h. Quantification of autophagy by counting the number of EGFP-LC3 dots in the transfected cells (see Materials and Methods) is shown on the right. Stars indicate statistically significant differences from mock-treated cells at ***p<0.005. Bar: 20 µm.

Autophagy is the other main proteolytic system in mammalian cells. Microtubule-associated protein 1 light chain 3 (LC3), an essential protein in the autophagic process, is considered the most appropriate marker of the autophagic flux [25]. The carboxyl-terminus of the precursor of this protein is cleaved to produce LC3-I (18 kDa), which is cytosolic and is converted to LC3-II (16 kDa) through conjugation with phosphatidylethanolamine and incorporation to the autophagosomal membrane. Therefore, to investigate the effect of PTEN expression on the autophagic flux we used again U87MG cells that express or not WT-PTEN or its mutants (C124S-PTEN and G129E-PTEN) and we monitored the LC3-II/actin ratios in the presence of lysosomal inhibitors to avoid the degradation of LC3-II in the autolysosomes [25]. As shown in Figure 4A, expression of WT-PTEN increased autophagy and this effect was lost in the cells expressing the C124S-PTEN mutant but not in those expressing the G129E-PTEN mutant. Since, in addition to LC3-II, LC3-I also increases its levels, it appears that the effect of PTEN on autophagy can be due to an enhanced expression of LC3. As an additional measurement of autophagy, the cells were transfected with an EGFP-LC3 construct and we observed higher amounts of fluorescent dots in the cells expressing WT-PTEN and its G129E mutant (Figure 4B). All these results indicate that in U87MG cells, expression of PTEN under the control of a tetracycline-inducible system increases autophagy independently of the lipid phosphatase activity of this protein.

**Figure 5:**
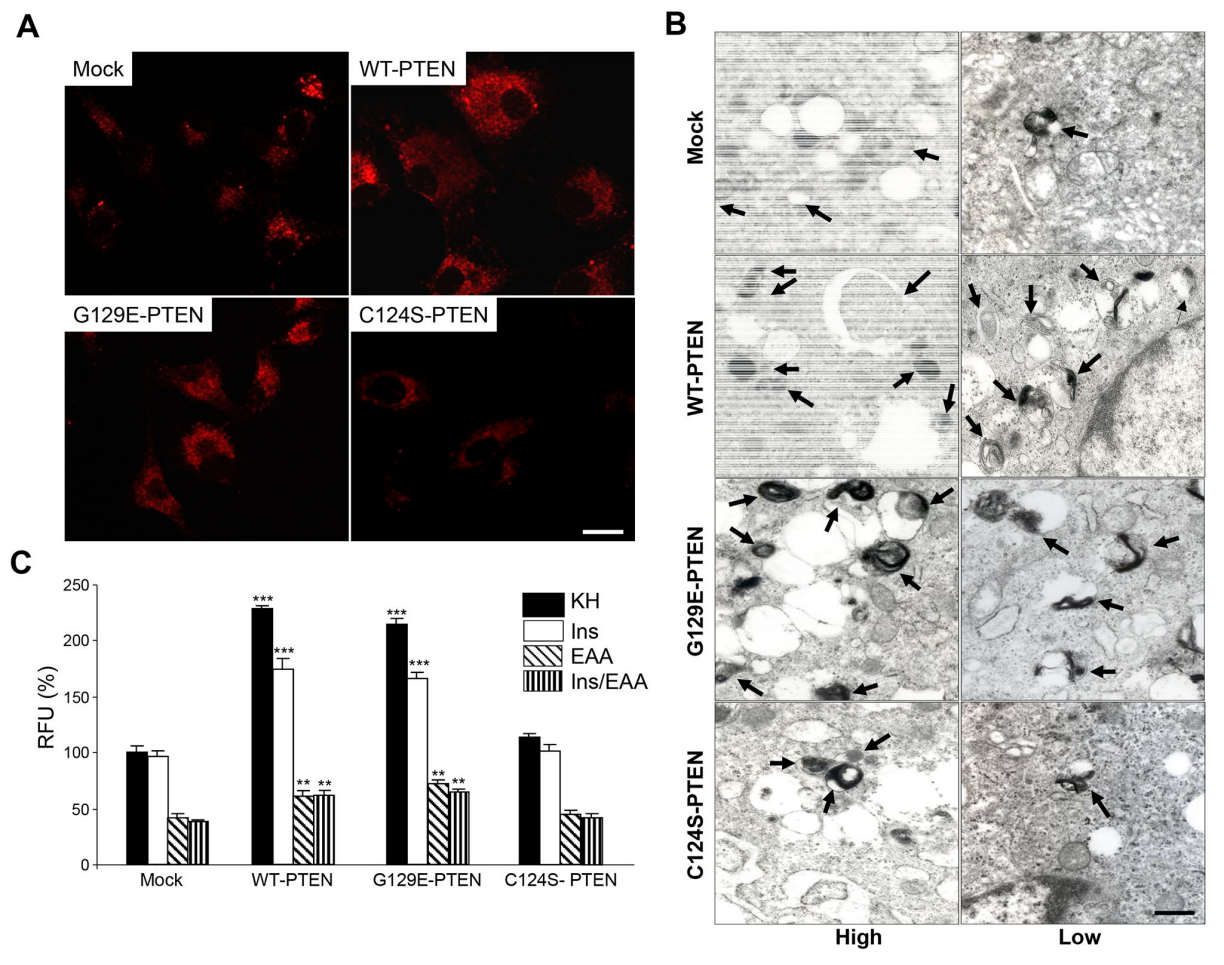
PTEN expression in U87MG cells increases the lysosomal mass. **A**) Representative fluorescence images of cells expressing WT-PTEN, C124S-PTEN and G129E-PTEN and of the mock-treated U87MG cells (control) incubated under high proteolysis conditions for 2 h and with LysoTracker Red (75 nM) during the last 15 min. Fluorescence is higher in cells expressing WT-PTEN and G129E-PTEN. Bar: 20 μm. **B**) Representative electron micrographs of the cells incubated for 2 h under high and low proteolysis conditions. Cells that express WT-PTEN and the G129E-PTEN mutant show more autophagic vacuoles (arrows) compared to the mock-treated cells or the cells expressing the C124S-PTEN mutant. Bar: 0.6 μm. **C**) Cells were incubated as in **A**) but under high (KH), intermediate (KH plus insulin, Ins, or amino acids, EAA) and low (KH plus insulin and amino acids, Ins/EAA) proteolysis conditions (see Materials and Methods). LysoTracker Red fluorescence in the cells was analyzed by flow cytometry as described in Materials and Methods. Relative fluorescence units (RFU) are expressed in percentage of the values obtained in the mock-treated U87MG cells incubated under high proteolysis conditions (KH). Results are the mean ± SD from seven independent experiments with duplicate samples. Stars indicate statistically significant differences from the corresponding (high, intermediate and low proteolysis conditions) values in mock-treated cells at **p<0.01 and ***p<0.005.

Autophagosomes fuse with endosomes/lysosomes to form autolysosomes with an acidic pH [26]. Therefore and since the lysosomal mass can be estimated by the fluorescence emitted from the acidophilic lysosomotropic probe LysoTracker Red, the same U87MG cells used before were incubated as above in the presence of this probe (Figure 5A). The results confirm the observations with the LC3 protein (see Figure 4): expression of WT-PTEN or its G129E-PTEN mutant increased the lysosomal mass when compared to the cells that express the catalytically inactive PTEN mutant (C124S-PTEN) and the mock-treated U87MG cells. Most probably, this increase occurs as a consequence of the augmented formation of autophagosomes. These results were confirmed by electron microscopy (Figure 5B): the number of autophagic vacuoles and lysosomes is higher in cells expressing WT-PTEN and its G129E mutant.

Next, we analyzed by flow cytometry the cells incubated as above with LysoTracker Red (Figure 5A), but also using KH containing separately insulin or amino acids (these two last conditions produce intermediate proteolysis in human fibroblasts, see [27]). Under all these conditions results were the same than before, namely increased lysosomal mass in WT-PTEN and in its G129E-PTEN mutant (Figure 5C, see first and last bar in each cell group). In addition, it was found that in U87MG cells, amino acids, but not insulin (except in the cells that express WT- or G129E-PTEN), decrease the lysosomal mass, and also that expression of WT-PTEN, or of its G129E mutant, increases the lysosomal mass under both conditions but especially in the cells incubated in KH plus insulin.

**Figure 6:**
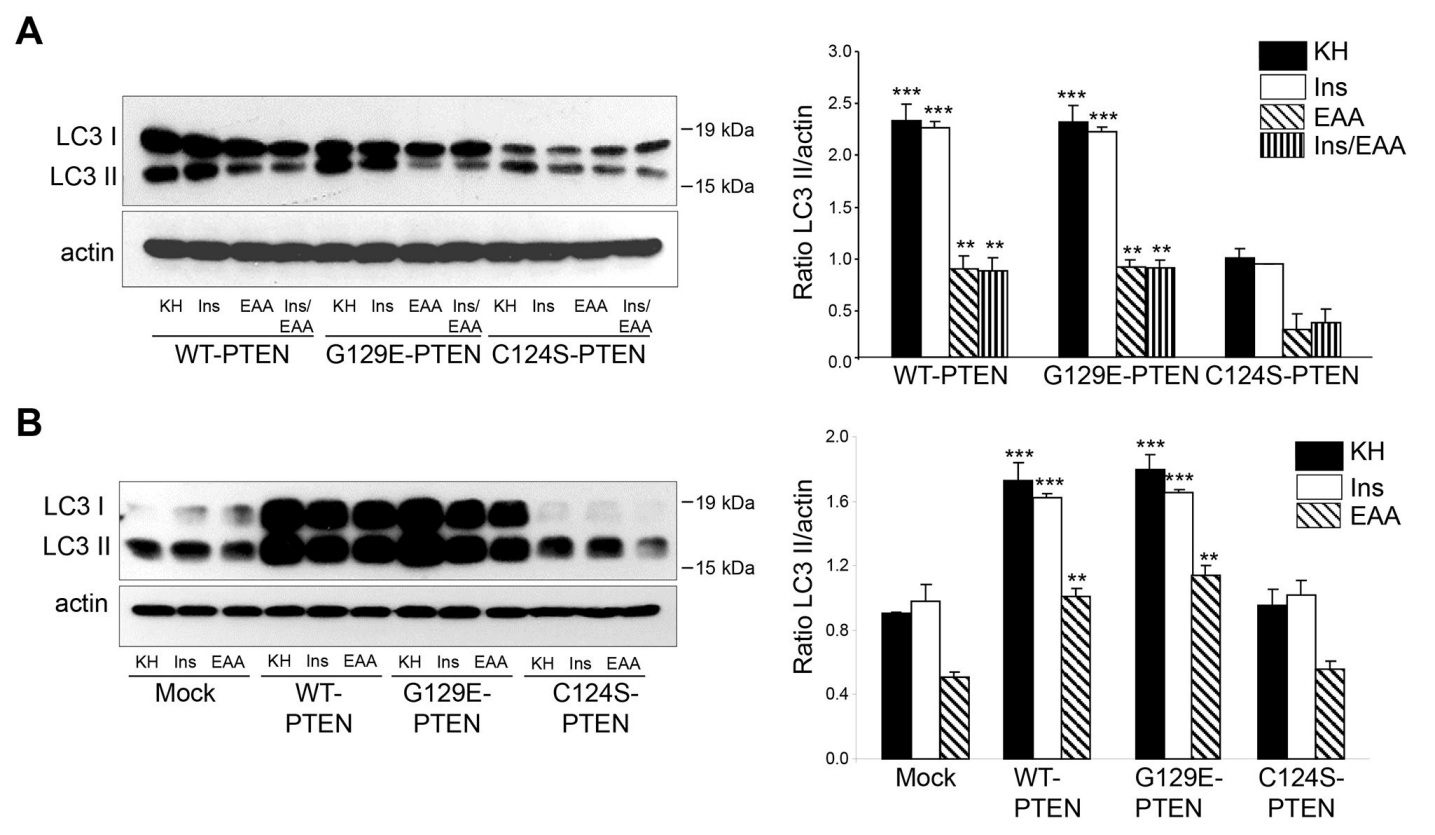
PTEN expression in U87MG cells increases the formation of autophagosomes under various proteolysis conditions. U87MG cells expressing WT-PTEN, G129E-PTEN or C124S-PTEN (**A**) and mock-treated U87MG cells or expressing WT-PTEN or C124S-PTEN (**B**) were incubated under high (KH), intermediate (Ins or EAA)(**A** and **B**) and low (Ins/EAA)(only in **A**) proteolysis conditions for 2 h as in Figure 5C in the presence of lysosomal inhibitors (100 µM leupeptin and 20 mM NH_4_Cl). Extracts (75 µg protein) were analyzed by SDS-PAGE and immunoblot with antibodies that recognize LC3 and, as a loading control, actin. The position of LC3-I and LC3-II bands are indicated on the left and molecular weight markers are indicated on the right. The Western blots on the left show representative experiments and the histograms on the right show the means ± SD of the densitometric analysis of the LC3-II/ratios from five different experiments. Stars indicate statistically significant differences from the corresponding (high, intermediate or low proteolysis conditions) values in the cells expressing the C124S PTEN mutant (**A**) and mock-treated cells (**B**) at **p<0.01 and ***p<0.005.

We also investigated separately the effects of insulin and amino acids on autophagosome formation in cells that express WT-PTEN and its G129E and C124S mutants and also in mock-treated cells, using the LC3 antibody. Therefore, the LC3-II/actin ratios were compared in these cells after 2 h incubation in the presence of lysosomal inhibitors under high (KH), intermediate (KH plus insulin or amino acids, separately) and low (KH plus insulin and amino acids) proteolysis conditions. As shown in Figure 6 (see also Figure 4A), expression of WT-PTEN and its G129E mutant increases the formation of autophagosomes when compared to the cells that express the C124S-PTEN mutant and the mock-treated cells. These effects were apparent even in the media that contain separately amino acids or insulin and with results comparable to those obtained when the lysosomal mass was analyzed (see Figure 5C). 

**Figure 7:**
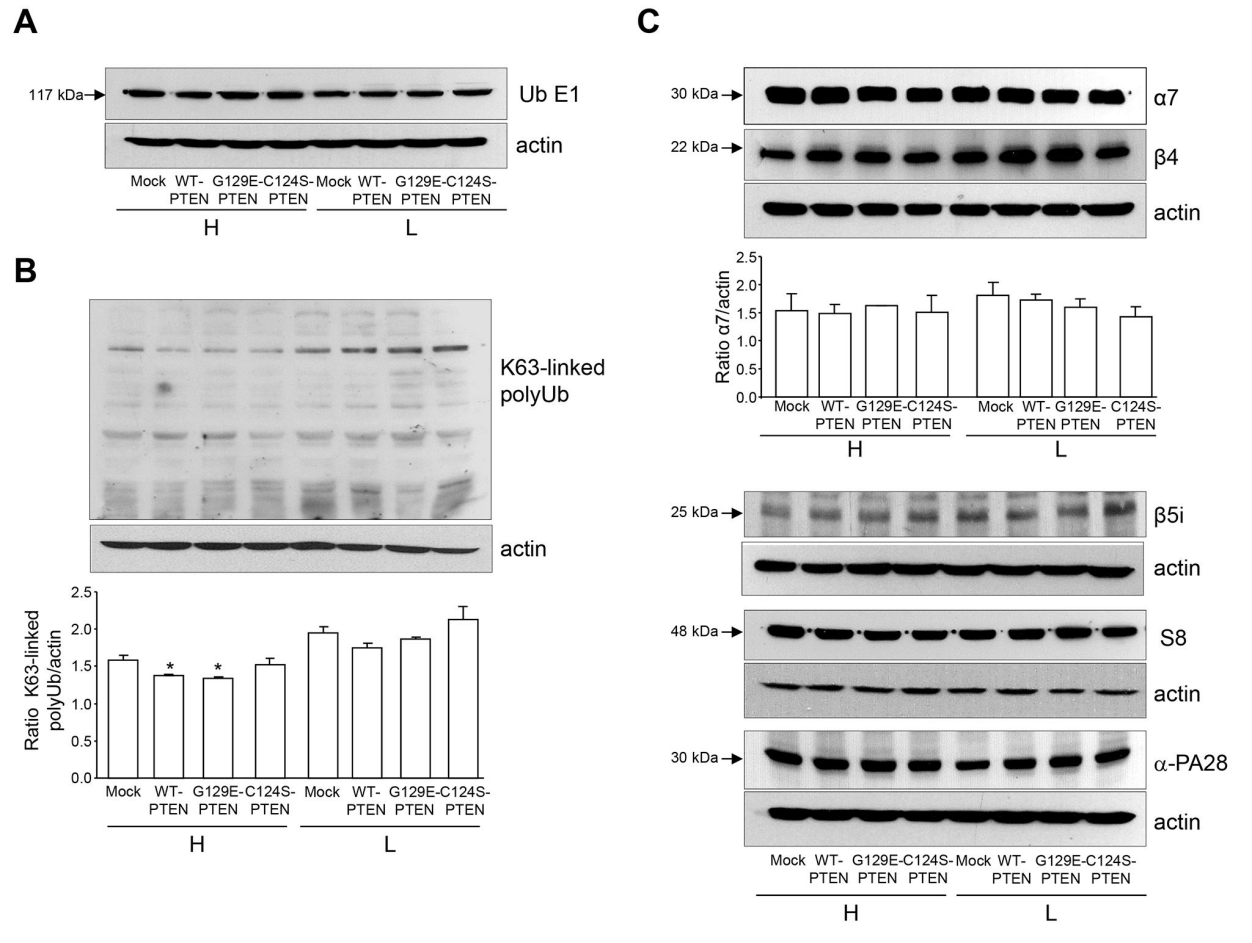
Levels of various components of the ubiquitin-proteasome system in U87MG cells expressing PTEN. U87MG cells expressing WT-PTEN, C124S-PTEN or G129E-PTEN and mock-treated (control) were incubated under high (H) or low (L) proteolysis conditions (see Materials and Methods) for 2 h. Total cell extracts (75 µg protein in A and C and 50 µg protein in B) were separated in SDS-PAGE and the gels were immunoblotted with antibodies raised against the ubiquitin activating enzyme E1 (**A**), K^63^-linked polyubiquitin chains (**B**) and the following proteasome subunits as indicated: α7 and β4 (20S proteasome), β5i (immunoproteasome), S8 (19S regulatory particle) and PA28α (11S regulatory particle)(**C**). Actin was also detected as a loading control. The figure shows representative gels from three different experiments. Relative levels of the bands were calculated by densitometric analysis and are only shown for K^63^-linked polyubiquitin chains (**B**) and α7 (**C**). Stars indicate statistically significant differences from mock-treated cells at *p<0.05 (in **B**). No significant differences were found in the other analyses.

In summary, all these results indicate that in the same cells in which autophagy increases (see Figures 4-6), the levels of ubiquitinated proteins (Figure 2) and the activity of proteasomes (Figure 3) decrease. Since autophagy is known to degrade ubiquitinated proteins [28] and even proteasomes [29], it could be possible that the induction of autophagy by PTEN observed here is responsible of the reduction in the activity of the UPS. Therefore, we investigated if the different cell lines used in these experiments have the same levels of: i) the ubiquitin-activating enzyme E1 (Figure 7A), ii) proteins with K63-linked polyubiquitin chains (Figure 7B), which are thought to be preferentially degraded by autophagy, and iii) various subunits of proteasomes and their regulatory complexes (Figure 7C). Results show that, except for the K63-linked ubiquitinated proteins under high proteolysis conditions, the levels of the other proteins were quite similar in the different cell lines. 

### PTEN expression in U87MG cells affects the PI3K class I/AKT/mTOR pathway through its lipid phosphatase activity

**Figure 8:**
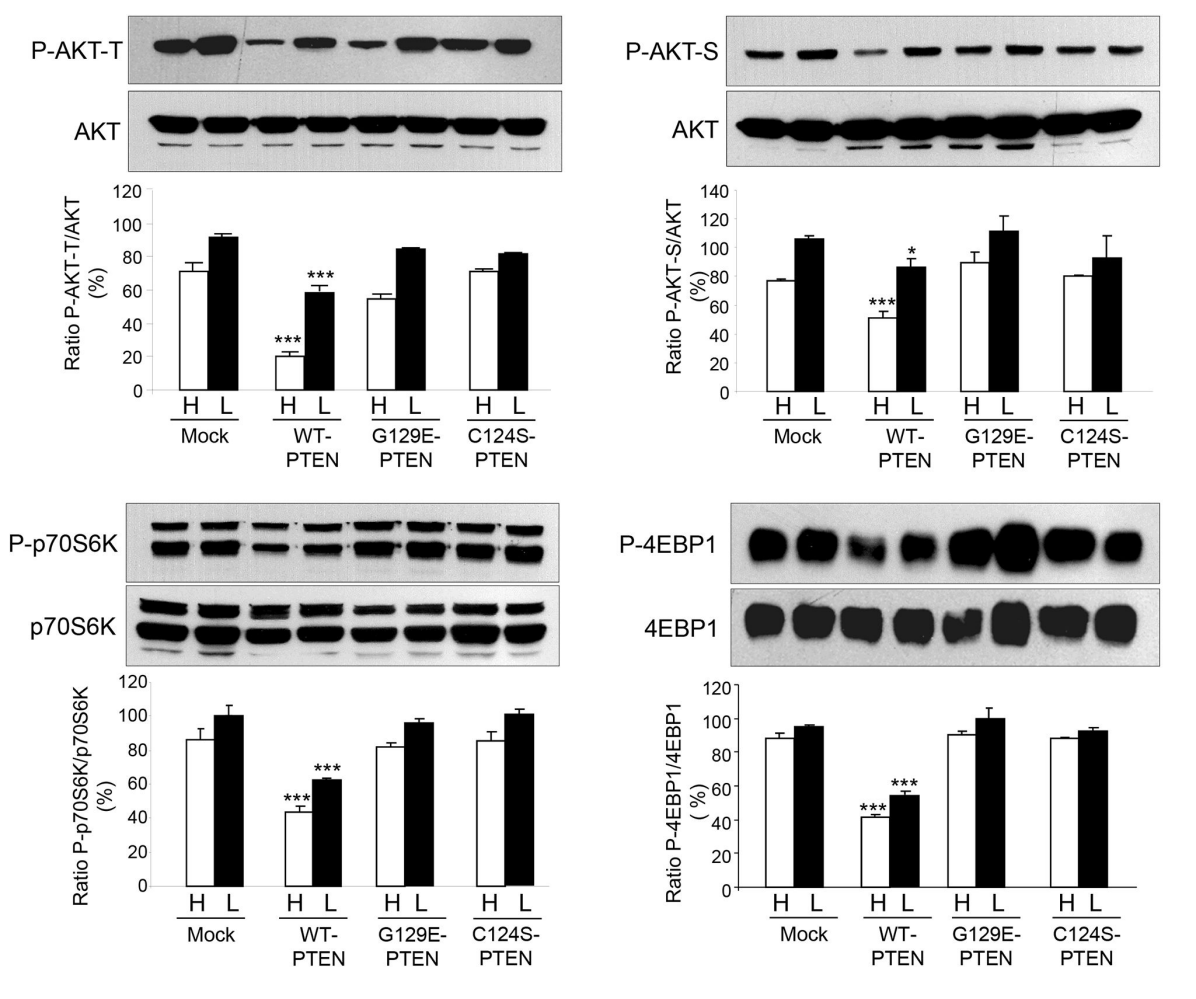
PTEN expression in U87MG cells affects the PI3K class I/AKT/mTOR signaling pathway. U87MG cells expressing WT-PTEN, C124S-PTEN or G129E-PTEN and mock-treated were incubated in high (H) and low (L) proteolysis media for 2 h. Total lysates were analyzed by Western blot using phospho-specific antibodies that recognize AKT-Thr308 (P-AKT-T) and AKT-Ser473 (P-AKT-S), p70S6K and 4-EBP1 and their respective pan-antibodies. The bands corresponding to the phosphorylated proteins, obtained from three different experiments, were densitometred and normalized to the corresponding total protein in the same sample. Results are shown on the histograms below the representative Western blots. Data are presented as percentage of the values in mock-treated (control) cells incubated in low proteolysis medium (L) and are means ± SD of three different experiments. Stars indicate statistically significant differences from the respective control values under high and low proteolysis conditions at *p<0.05 and ***p<0.005.

PTEN is a dual phosphatase that negatively regulates the PI3K class I/AKT/mTOR pathway by dephosphorylating the product of PI3-Kinase class I, phosphatidylinositol (3,4,5)-trisphosphate [30]. Therefore, we investigated if expression of PTEN in U87MG cells affects this signaling pathway by its lipid phosphatase activity. Immunoblot analysis shows that the phosphorylation of three relevant proteins of this signaling pathway, AKT and two mTOR substrates, p70S6K and 4-EBP1, decreased as expected when WT-PTEN was expressed (Figure 8). Figure 8 also shows that these effects were not produced when any of both PTEN mutants (C124S or G129E) was expressed. Thus, as in other cells, expression of WT-PTEN in U87MG cells negatively regulates the PI3K class I/AKT/mTOR pathway through its lipid phosphatase activity.

### Inhibition of the ubiquitin-proteasome system and activation of autophagy by PTEN expression is independent of m-TOR

**Figure 9:**
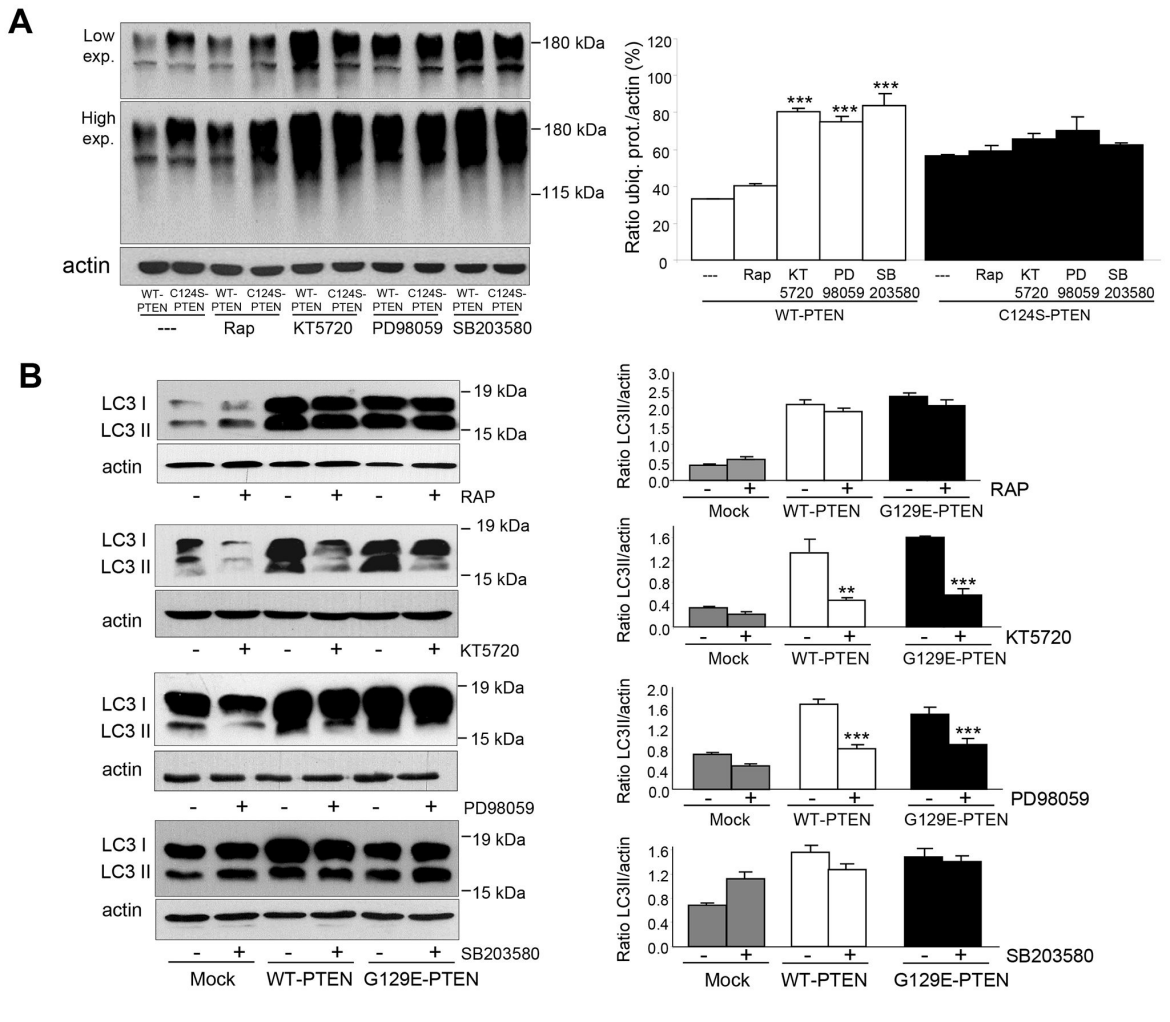
Inhibition of the ubiquitin-proteasome pathway and activation of autophagy by PTEN in U87MG cells are independent of mTOR. U87MG cells expressing WT-PTEN or C124S-PTEN (**A**) or WT-PTEN or G129E-PTEN and mock-treated (**B**) were incubated for 18 h with doxycycline and in the last 2 h the following inhibitors were added as indicated: rapamycin (RAP, 200 mM, mTOR inhibitor), KT5720 (25 µM, PKA inhibitor), PD98059 (10 µM, ERK1/2 inhibitor) and SB203580 (10 µM, p38 inhibitor). Before collecting the cells, proteasome (50 µM MG132, **A**) and lysosomal (100 µM leupeptin and 20 mM NH_4_Cl, B) inhibitors were also added for 1 h. Total lysates were analyzed by SDS-PAGE and Western blot with antibodies that recognize ubiquitinated proteins (FK1) (**A**), LC3 (**B**) and, as a loading control actin. Molecular weight markers are indicated on the right and in **B** the position of LC3-I, and LC3-II bands are also shown. The histograms on the right show means ± SD of the densitometric measurements from three different experiments and are expressed as amounts of ubiquitinated proteins (ubiq. prot.) normalized to the levels of actin (**A**) or as LC3II/actin ratios (**B**). Stars indicate statistically significant differences from the values without the corresponding inhibitor treatment at **p<0.01 and ***p<0.005.

Expression of WT-PTEN decreases the levels of ubiquitinated proteins and this effect is lost when the C124S-PTEN mutant is expressed instead of WT-PTEN (see Figure 2). To further investigate if the effects produced by PTEN occur *via* mTOR, we compared these effects in U87MG cells that express WT-PTEN or its C124S-PTEN mutant in the presence or not of the specific mTOR inhibitor rapamycin. As shown in Figure 9A, inhibition of mTOR did not modify the effect produced by WT-PTEN on the ubiquitination of proteins. In contrast, these effects were reduced to some extent in the presence of inhibitors of other kinases that can affect intracellular protein degradation, such as KT5720, PD98059 and SB203580 targeting, respectively, protein kinase A (PKA), ERK1/2 and p38 MAPK. We also investigated the effect of PTEN on autophagy (Figure 9B) comparing mock-treated cells with the cells expressing WT-PTEN or its G129E mutant. Again, rapamycin was without effect while this activation was lost in the presence of inhibitors of PKA and ERK1/2. Therefore, it appears that PTEN can also regulate autophagy by other pathways different from the phosphatidylinositol 3-kinase class I/AKT/mTOR signaling pathway.

## Discussion

Autophagy and the UPS, the two main mechanisms of intracellular protein degradation, have been usually considered independent from each other because of their differences in degradation mechanisms, physiological significance, substrate proteins and signals stimulating or inhibiting them [1]. In addition, autophagy can also degrade polysaccharides, nucleic acids, lipids and other molecules that are not substrates of the UPS. However, more recently it has become apparent that these two mechanisms are mechanistically linked and that their regulation and activities can proceed in concert [20-22]. 

Both UPS and autophagy have been associated with cancer development and their modulation has been proposed as a target for cancer therapy [31, 32]. In fact, various oncoproteins and tumor suppressors, which are substrates of the UPS, regulate autophagy [9,10]. Here, we have evaluated the role of one of these tumor suppressors, PTEN, in the regulation of UPS and autophagy in U87MG human glioma cells that lack a functional PTEN. We used these cells instead of others expressing PTEN to avoid interferences from the endogenous protein with the expression of exogenous WT or mutant PTEN proteins (or *viceversa*) and to mimic the loss of PTEN in tumours. 

PTEN is known to be a substrate of the UPS in cancer cell lines *via* various E3 ubiquitin ligases, including Nedd4 [33]. This protein has also been found to be negatively regulated by its substrate PTEN through its lipid phosphatase activity [34] that antagonizes the phosphorylation of AKT by PI3K. Our results indicate that expression of WT-PTEN in U87MG glioma cells caused a decrease in both the amount of ubiquitinated proteins and in the chymotrypsin-like peptidase activity of proteasomes. This decrease was not associated with the lipid phosphatase activity of PTEN, thus suggesting a new function for the protein phosphatase activity of PTEN.

**Figure 10:**
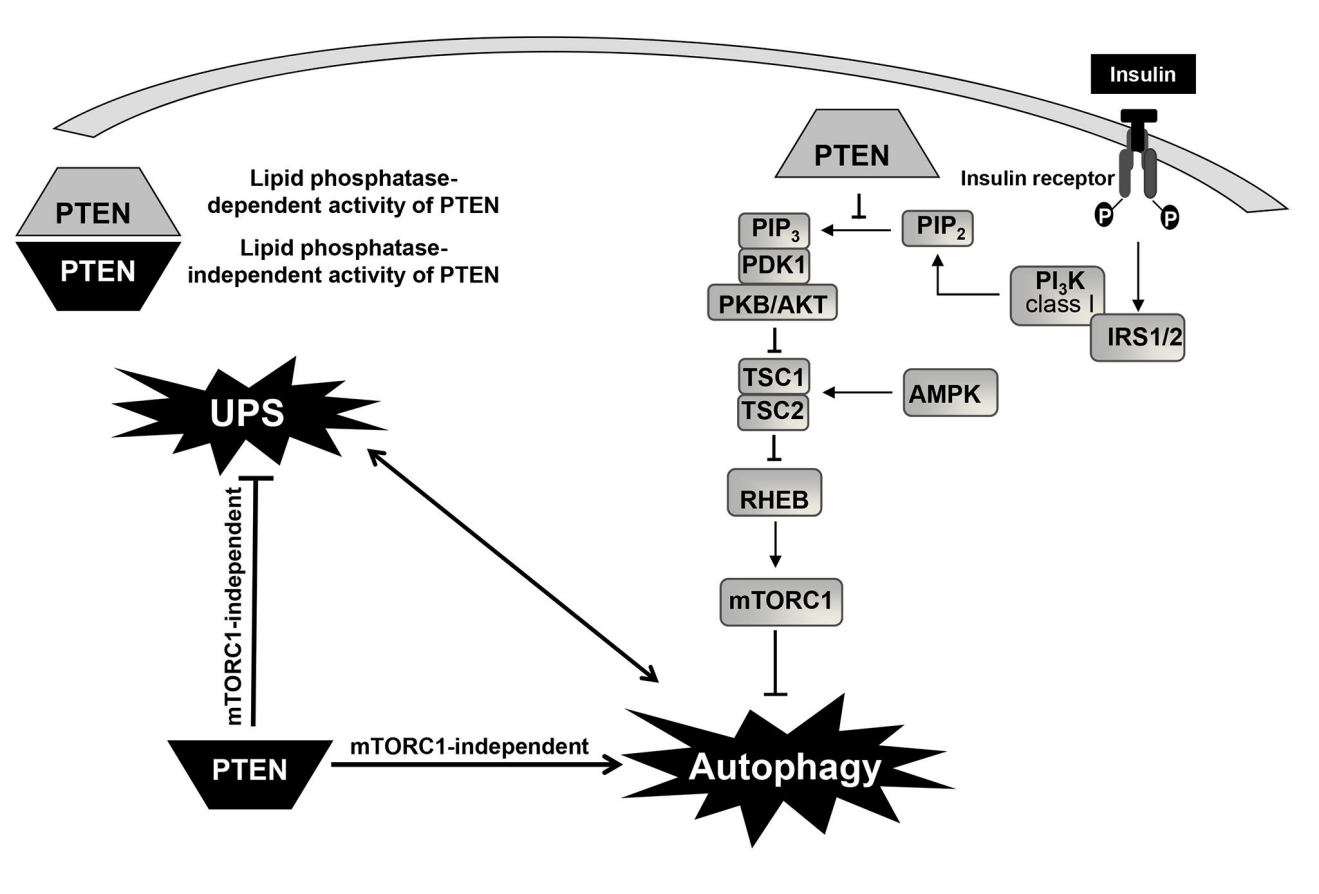
Lipid phosphatase-dependent and -independent roles of PTEN in the regulation of autophagy. In a simplified scheme, insulin signalling to mTOR complex 1 (mTORC1) is mediated through phosphorylation of the insulin receptor 1 or 2 (IRS 1/2), subsequent activation of PI_3_K class I and protein kinase B/AKT, which either directly, or indirectly *via* inhibition of the tuberous sclerosis complex 1 and 2 (TSC1/TSC2) and activation of the small GTPase RHEB, activates mTORC1 (an inhibitor of autophagy). Adenosine 5-monophosphate-activated protein kinase (AMPK) can inhibit mTOR indirectly after activation of TSC1/TSC2 by phosphorylating residues different from those phosphorylated by AKT. PTEN, through its lipid phosphatase activity inhibits the generation of phosphatydilinositol (3,4,5)-trisphosphate (PIP_3_) from phosphatydilinositol (3,4)-bisphosphate (PIP_2_) caused by the above mentioned activation of class I PI3K. This leads to the inhibition of AKT/PKB signalling, which activates the TSC1/2 complex, restrains mTORC1 and consequently induces autophagy. By contrast, in U87MG cells, expression of PTEN under the control of a tetracycline-inducible system activates autophagy and inhibits the ubiquitin-proteasome system (UPS) through a lipid phosphatase-independent activity and in an mTOR-independent way. ERK1/2 and PKA may be involved in this signaling pathway. In contrast to autophagy, much less is known on the regulation of the ubiquitin-proteasome system. Both catabolic pathways are closely interrelated (indicated by the double headed arrow interconnecting them) and, therefore, it could be also possible that PTEN directly affects only one of them.

Concerning autophagy, tumor suppressors affect cancer development in different and complex ways [35] and the autophagic response to cancer seems to depend on its progression stage [36,37]. Most tumor suppressors promote autophagy, which has been suggested as a potential mechanisms to inhibit tumor development [38], and only few of them, such as BRCA1 [39], produce the opposite effect. PTEN is known to dephosphorylate phosphatydilinositol (3,4,5)-trisphosphate and, consequently, to inhibit the PI3K class I/AKT/mTOR signaling pathway, which is mainly activated by insulin [40]. In fact, expression of PTEN in human colon cancer HT-29 cells increases autophagy *via* this signaling pathway [19]. Furthermore, at least another study [41] in hepatocytes in which autophagy was already inhibited by insulin showed that loss of PTEN function increased this inhibition without affecting LC3 lipidation. The results obtained here in U87MG cells are consistent with both reports regarding the described increase of autophagy produced by PTEN. However, they differ from both in the mechanism of activation of autophagy, which according to our data does not depend on the lipid phosphatase activity of PTEN and occurs with increased LC3-II levels. PTEN expression in U87MG cells inactivates the PI3K class I/AKT/mTOR signaling pathway by its lipid-phosphatase activity, as shown by the decreased phosphorylation of AKT and of two mTOR substrates in both mutants. Although previous studies have reported that interruption of the PI3K class I/AKT/mTOR pathway with siRNAs or pharmacological inhibitors can induce autophagy in human glioma cells [42,43], we observed that under our conditions insulin does not inhibit autophagy in the mock-treated U87MG cells. Therefore, it is possible that PTEN deficiency in these cells renders their autophagic response insensitive to the PI3K class I/AKT/mTOR signaling pathway, which is mainly activated in other cells by insulin. Thus, in addition to the established role of the lipid phosphatase activity of PTEN in activating autophagy *via* the PI3K class I/AKT/mTOR signaling pathway, this tumour suppressor can also activate autophagy by other pathways that require LC3 lipidation and that are independent of its lipid phosphatase activity (Figure 10). Although some protein substrates of PTEN, such as CREB, FAK, SHC or PDGFR and PTEN itself, have been documented [44], little is known on the specific sites of these dephosphorylations and on the mechanistic details. Using specific inhibitors of various pathways, our results show that PTEN can activate autophagy *via* other signaling pathways independently of mTOR. However, much more experiments would be necessary to identify all the molecular details of the specific pathway.

In summary, our study has demonstrated that in U87MG glioma cells PTEN expression increases autophagy and decreases the activity of the UPS. In addition, these effects occur independently of the lipid-phosphatase activity of PTEN. Since these two proteolytic systems are closely related to cancer development, identification of the mechanisms by which the tumor suppressor PTEN can regulate them could allow the identification of new therapeutic targets for cancer treatment.

## Materials and Methods

### Materials

Dulbecco's Modified Eagle´s Medium (DMEM), Minimum Essential Medium (MEM), MEM non-essential amino acids (NEAA), MEM essential amino acids (EAA), foetal bovine serum, glutamine, sodium pyruvate and penicillin/streptomycin were supplied by Invitrogen Life Technologies. Trypsin-EDTA, hygromycin B, doxycycline, human insulin, EDTA, NH_4_Cl, dithiothreitol and phenylmethylsulfonyl fluoride (PMSF) were purchased from Sigma-Aldrich. Leupeptin and N-Succinyl-Leu-Leu-Val-Tyr-7-amino-4-methylcoumarin (N-Suc-LLVY-MCA) were from Peptide Institute, Inc. and LysoTracker Red from Molecular Probes-Invitrogen Life Technologies. The inhibitors rapamycin, KT5720, PD98059 and SB203580 were from Calbiochem-Merck4Biosciences. Phospho-specific antibodies that recognize AKT (Thr308 and Ser 473), 4E-BP1 (Thr37 and Thr46) and p70S6K (Ser434) and their corresponding pan antibodies, as well as an antibody that recognizes PTEN, were from Cell Signalling. Antibodies against LC3 were from Nanotools and antibodies that recognize polyubiquitinated (FK1) and mono- and polyubiquitinated (FK2) proteins, but not free ubiquitin, were from Abcam. The antibodies that recognize the ubiquitin activating enzyme E1, polyubiquitin (K^63^-linkage specific) and the proteasome subunits α7 and β4 (20S proteasome), LMP7 (immunoproteasome), S8 (19S regulatory particle) and PA28α (11S regulatory particle) were from Enzo Life Sciences. Horseradish peroxidase-conjugated anti-rabbit and anti-mouse IgGs antibodies were from Sigma-Aldrich. Bovine serum albumin, G-418 (geneticin), X-tremeGENE 9 DNA Transfection Reagent, polyvinylidene difluoride (PVDF) membranes and Lumi-Light Western Blotting Substrate were from Roche España. The pEGFP-LC3 plasmid was kindly provided by Dr. T. Yoshimori (Osaka University, Japan). All the remaining reagents were of the highest purity available.

### Cell culture

All cells were grown at 37°C in a 5% (v/v) CO_2_ humidified incubator. Human glioma U87MG cells were purchased from the European Collection of Animal Cell Cultures (UK, Cat. No. 89081402). Cells were grown in DMEM supplemented with 10% foetal bovine serum, 2 mM L-glutamine, 1 mM sodium pyruvate, 1% non-essential amino acids, 100 units/ml penicillin and 100 µg/ml streptomycin. U87MG cells were transfected with the Tet-on 3G inducible vector from Clontech, either empty (mock-treated cells) or containing the appropriate constructions (see below), using X-tremeGENE 9 DNA Transfection Reagent, following the manufacturer’s instructions. Besides mock-treated cells (containing the empty vector), the other cell lines contained the same vector, but expressing one of the following proteins: wild type PTEN (WT-PTEN) or PTEN with the G129E mutation (G129E-PTEN, PTEN without lipid phosphatase activity but with protein phosphatase activity) or the C124S mutation (C124S-PTEN, PTEN without both, lipid and protein, phosphatase activities). All the PTEN constructions were sequenced using an ABI-3730xl DNA Analyzer (Applied Biosystems) and confirmed to be correct. The cell lines were grown in the medium described above, but supplemented with 300 μg/ml G-418 and 75 μg/ml hygromycin B. To induce the expression of PTEN and of its mutants, doxycycline (1 µg/ml) was added for 18 h. Krebs-Henseleit medium (KH: 118.4 mM NaCl, 4.75 mM KCl, 1.19 mM KH_2_PO4, 2.54 mM MgSO_4_, 2.44 mM CaCl_2_.2H_2_O, 28.6 mM NaHCO_3_, 20 mM glucose), containing 10 mM Hepes, pH 7.4, was used to induce high proteolysis conditions. To obtain, respectively, intermediate or low proteolysis conditions, insulin (0.1 mM) or EAA (at two times the concentration present in the growth medium) separately, or insulin and EAA together were added to the KH [23]. In some cases, the full growth medium was used instead of the KH plus insulin and EAA to produce low proteolysis conditions with similar results. Induction of autophagy was also assessed in cells transfected with pEGFP-LC3 by counting the number of GFP-LC3 dots per cell in a minimum of 20 random cells per condition and experiment as described previously [45]. All procedures with the cells used in this study were approved by the CIPF Ethics Committee.

### Proteasome activity

The different U87MG cells were incubated for 2 h in high or low proteolysis media, scrapped in lysis buffer (50 mM Hepes, pH 7.4, 50 mM EDTA, 50% glycerol, 100 mM dithiothreitol) and subjected to ten cycles of freezing (-80°C) and thawing (37°C). Cell homogenates were centrifuged at 10,000 *g* for 10 min at 4°C and the chymotrypsin-like activity of proteasomes was determined in the clarified supernatants as described previously [46]. Briefly, 10 μg of proteins were used in a final volume of 200 μl for each reaction. The fluorogenic substrate N-Suc-LLVY-AMC (2 mM) was added to each well and the plate was incubated in the dark at 37°C. Measurements were carried out in a Spectra Max M5 microplate reader (Molecular Devices) at excitation and emission wavelengths of 380 and 460 nm, respectively. The chymotrypsin-like activity of proteasomes was determined at the indicated times as the difference between total activity and the activity that remains after incubation with the specific proteasome inhibitor lactacystin (40 µM).

### Flow cytometry

After induction with doxycycline, cells were incubated in KH or in full growth medium for 1 h 45 min. Cells were then detached with trypsin-EDTA and resuspended in phosphate buffered saline (PBS). To determine the lysosomal mass, the cells (10^6^ cells/ml) were incubated in suspension with 75 nM LysoTracker Red for 15 min at 37°C and the emitted red fluorescence (620 ± 20 nm band-pass filter) was measured (10^5^ cells per sample) by flow cytometry as described [39], using a Cytomics FC 500 Flow cytometer (Beckman Coulter).

### Western blot

Western blot was carried out essentially as described [47]. Briefly, whole cell extracts were prepared in ice-cold lysis buffer (50 mM Tris-HCl, 2 mM EDTA, 150 mM NaCl, 1% Nonidet P-40, 15% glycerol, 1 mM PMSF, 0.1 mM leupeptin, 1 mM Na_3_VO_4_, 2 mM NaF, 2 mM Na_4_PO_7_, pH 7.4 ). Proteins (75 μg) were separated by SDS/PAGE and transferred to PVDF membranes, which were blocked with 5% dry milk in Tris-buffered saline (10 mM Tris-HCl, pH 7.4, 150 mM NaCl) for 1 h at 20°C. Next, membranes were first incubated with the appropriate primary antibodies (anti-LC3 and anti-S8 were used at a 1:500 and 1:2000 dilutions, respectively, the rest at 1:1,000) for 1 h at 20°C or overnight at 4°C, and then with the corresponding horseradish peroxidase-conjugated secondary antibodies (1:5,000 dilution) for 1 h at 20°C. The blots were developed using standard chemiluminiscence (Roche España). Protein bands were quantified by densitometric analysis with an Image Quant ECL (GE Healthcare).

### Fluorescence and electron microscopy

Fluorescence microscopy was carried out as previously described [48]. Briefly, cells grown on coverslips were incubated with 75 nM Lysotracker Red for 15 min at 37°C. Then, the cells were rinsed with PBS, fixed with 3.7% formaldehyde/PBS for 15 min at 37°C, washed again with PBS, mounted using Fluor-Save reagent (Calbiochem) and observed with an Apotome-equipped Axio Observer Z1 microscope (Carl Zeiss AG).

Electron microscopy was performed essentially as described [49]. Briefly, cells were prefixed with 2% glutaraldehyde in 0.1 M PIPES buffer (pH 7.3) at 4°C and washed and postfixed with 1% OsO_4_ in 0.05 M acetate veronal buffer at 4°C. After dehydration in acetone and embedding in Epon, ultra-thin sections were stained with lead citrate for observation under a Philips 300 electron microscope.

### General procedures

Protein concentrations were determined using a BCA Protein Assay Kit (Pierce), according to the manufacturer's instructions, or a Lowry-deoxycholate method [50]. All experiments were performed at least three times. Data are means ± SD. Statistical significance of the data was evaluated by factorial ANOVA test using Graph Pad Prism (version 5.0) statistical software. Differences were considered significant at *p< 0.05; **p< 0.01; and ***p< 0.005.
